# *Lactobacillus plantarum* QL01 Alleviates D-Galactose-Induced Oxidative Stress and Restores Gut Microbiota in Ageing Mice

**DOI:** 10.3390/nu18010035

**Published:** 2025-12-21

**Authors:** Haichuan Li, Mingqing Zhang, Diyan Wu, Di Gong, Jiazhang Huang, Zhenchuang Tang, Liang Wang, Ying Zhang

**Affiliations:** 1 School of Public Health, Lanzhou University, Lanzhou 730000, China; 2 Institute of Food and Nutrition Development, Ministry of Agriculture and Rural Affairs, Beijing 100000, China; 3 Department of Public Health, Global Health Institute, Marshall University, Huntington, WV 25703, USA

**Keywords:** *Lactobacillus plantarum* QL01, oxidative stress, gut microbiota

## Abstract

Background/Objectives: This study aimed to evaluate the in vivo antioxidant effects of *Lactobacillus plantarum* QL01 and to provide a theoretical basis for the use of probiotics in alleviating conditions associated with oxidative stress. Methods: A D-galactose-induced aging model was established in fifty 8-week-old SPF male Kunming mice, which were randomly allocated into five groups: normal control (NC), model control (MC), positive control (VC, ascorbic acid 200 mg kg^−1^ day^−1^), low-dose bacterial (LP, 1 × 10^9^ CFU kg^−1^ day^−1^), and high-dose bacterial (HP, 1 × 10^10^ CFU kg^−1^ day^−1^) groups. Except for the NC group, all mice received the daily intraperitoneal injection of D-galactose (125 mg kg^−1^ day^−1^) for 8 weeks to induce oxidative stress. Corresponding treatments or equal volumes of saline were administered daily by gavage. Results: After 8 weeks, serum, liver, colon, and fecal samples were collected and analyzed to evaluate the efficacy of QL01 in counteracting oxidative stress and restoring gut microbiota homeostasis. The results demonstrated that tissue atrophy and the levels of various inflammatory factors, including interleukin-6 (IL-6), interleukin-1β (IL-1β), tumor necrosis factor-α (TNF-α), and interleukin-10, were inhibited (*p* < 0.05). It was further demonstrated that the levels of total antioxidant capacity (T-AOC), superoxide dismutase (SOD), glutathione (GSH), catalase (CAT), and malondialdehyde (MDA) were significantly reversed in hepatic tissues by QL01 intervention (*p* < 0.05), thereby leading to the alleviation of inflammatory responses and oxidative stress in ageing mice. Pathological observations revealed that QL01 mitigated the damage to liver and colon tissues in mice. In addition, the intervention of QL01 led to an improvement in the expression of tight junction proteins in the colonic tissues of mice, as determined by qPCR. Additionally, the host’s intestinal microbiota and metabolites were restored. Conclusions: Overall, this study revealed that *Lactobacillus plantarum* QL01 is a promising candidate for modulating oxidative stress and the homeostasis of the host’s gut flora.

## 1. Introduction

Free radicals, inevitable byproducts of aerobic metabolism, are tightly regulated by the intrinsic antioxidant system under physiological conditions [1]. However, ageing, environmental toxins, or metabolic disorders disrupt the balance between their generation and scavenging, leading to excessive accumulation, oxidative stress, and subsequent cellular damage (e.g., lipid peroxidation, protein modification, nucleic acid fragmentation) [2,3]. Notably, oxidative damage caused by free radical-mediated stress harms cell membranes, mitochondria, and other cellular structures, ultimately leading to impaired function or cell death via apoptosis. This process contributes to a wide range of pathologies, including aging, cancer, and cardiovascular disease.

D-galactose is a natural nutrient for the human body. However, excess D-galactose can be converted into galactitol, which persistently induces the production of reactive oxygen species. This can lead to increased malondialdehyde activity and reduced activity of antioxidant enzymes such as glutathione peroxidase and superoxide dismutase [4]. This, in turn, can lead to inflammation, oxidative stress, and organ dysfunction [5]. Accumulating evidence has demonstrated that probiotics serve as a promising class of dietary antioxidants [6]. Among them, lactic acid bacteria (LAB), commonly found in fermented foods and widely consumed, represents a major group of probiotics with multiple documented health benefits [7]. Notably, many studies have demonstrated that LAB possesses strong potential for alleviating oxidative stress, including increased the activities of GSH, CAT and SOD in brain and liver tissues of aging mice [8]. However, a critical characteristic is that the antioxidant capacity of LAB is highly strained specifically. This variability has been consistently observed across different studies. For instance, Lin et al. (2000) reported that *Bifidobacterium longum* 15708 exhibited higher free radical-scavenging activity than *Lactobacillus acidophilus* 4356 [9]. Similarly, Shen et al. (2011) found a significant difference in the free radical scavenging capacity between different fractions of *Bifidobacterium animalis* 01 [10]. More recently, a systematic screening of 1205 LAB isolates from the Qinghai–Tibet Plateau further confirmed that their antioxidant properties vary not only at the species level but also across different cellular fractions [11].

It is well established that gut microbiota plays a vital role in maintaining host health. D-galactose alters oxidative stress levels, which subsequently affects the intestinal microenvironment and leads to gut microbiota dysbiosis [12]. Accumulating evidence indicates that the gut microbiota significantly influences the host’s redox balance. An abnormal gut microbiota dominated by harmful bacteria can trigger oxidative stress. In turn, oxidative stress disrupts the structure and diversity of the gut microbiota, resulting in dysbiosis, alterations in the Firmicutes/Bacteroidetes (F/B) ratio, reduced relative abundance of beneficial bacteria, and increased relative abundance of potential pathogenic bacteria [13]. Currently, probiotic modulation represents a promising therapeutic approach [14]. Supplementation with probiotics can counteract oxidative stress by restoring gut microbiota balance, thereby effectively alleviating aging-related symptoms [15].

Our research has demonstrated the antioxidant properties of *Lactobacillus plantarum* QL01 in vitro [11]. However, the mechanism underlying QL01’s antioxidant activity in the host remains unclear. Hence, this study focuses on the antioxidative capacity of QL01 and its ability to alleviate oxidative damage induced by D-galactose in ageing mice. Additionally, the gut microbiota analysis is performed to improve our understanding of the mechanisms. This study could provide valuable theoretical insights and a reliable strain resource for the development of antioxidant and anti-ageing probiotic products.

## 2. Materials and Methods

### 2.1. Strain Source and Culture

*Lactobacillus plantarum* QL01 (CCTCC NO: M 20241615), previously isolated from yak milk on the Qinghai–Tibet Plateau, exhibits antioxidant capacity. It was cultured in de Man, Rogosa, and Sharpe (MRS) liquid medium (OXOID Biotechnology Ltd., Basingstoke, UK) at 37 °C for 18–24 h under aerobic conditions.

### 2.2. Preparation and Verification of the Bacterial Dose

For gavage preparation, a fresh culture of *L. plantarum* QL01 was inoculated into sterile MRS broth and incubated statically at 37 °C for 24 h. Bacterial cells were harvested by centrifugation at 10,000 rpm for 15 min at 4 °C. The precipitate was collected and washed twice with PBS (pH = 7.4) and then resuspended in sterile PBS. The optical density at 600 nm (OD_600_) of the suspension was measured. Its concentration was adjusted to the target values of 10^9^ CFU mL^−1^ and 10^10^ CFU mL^−1^ by referring to a pre-established standard curve (correlating OD_600_ with viable counts). To confirm the accuracy of the prepared doses, the bacterial suspension was serially diluted in PBS, spread on MRS agar plates, and incubated anaerobically at 37 °C for 48 h. The colony-forming units (CFU) were counted, which validated that the administered doses were 1 × 10^9^ CFU kg^−1^ day^−1^ (low-dose group) and 1 × 10^10^ CFU kg^−1^ day^−1^ (high-dose group), given in a 0.2 mL gavage volume. Fresh suspensions were prepared immediately before each daily administration.

### 2.3. Animal Experiment Design

**Figure 1:**
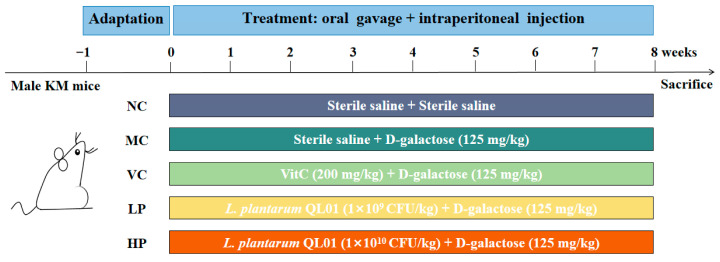
Experimental flowchart of animal treatments.

Fifty SPF-grade Kunming mice (male, eight weeks old, weighing 18–24 g) were purchased from the Laboratory Animal Center of Lanzhou University. The mice were group-housed (5 per cage) in positive-pressure isolation cages (BioZone Ltd., Ramsgate, Kent, United Kingdom) with corn cob bedding, Wooden chew blocks were provided as environmental enrichment. The housing environment was maintained at 22 ± 2 °C and 55 ± 10% relative humidity under a 12 h/12 h light-dark cycle. All mice were allowed free access to standard chow diet and autoclaved water. Cage cleaning was performed twice weekly to ensure hygiene. After one week of adaptation, the mice were randomly divided into five groups (n = 10): normal control (NC), model control (MC), positive control (VC), low-dose QL01 (LP), and high-dose QL01 (HP). The mice in the MC, VC, LP, and HP groups were intraperitoneally injected with D-galactose saline solution (Sigma-Aldrich Corporation, St. Louis, MO, USA) at a dose of 125 mg kg^−1^ day^−1^. In contrast, the NC group received an equivalent volume of sterile saline. The VC group was additionally administered ascorbic acid at a dose of 200 mg kg^−1^ day^−1^. The LP and HP groups were treated with QL01 saline solution at a dose of 1 × 10^9^ CFU mL^−1^ day^−1^ and 1 × 10^10^ CFU mL^−1^ day^−1^, respectively. All intraperitoneal injections and oral gavages were performed at a volume of 0.2 mL per mouse, with the actual administered amount adjusted weekly based on individual body weight. The experiment lasted for 8 weeks (Figure 1).

At the end of the 8 weeks, blood samples were collected as a terminal procedure prior to euthanasia. Mice were first deeply anesthetized with isoflurane. Terminal blood was then rapidly collected from the retro-orbital (eye) puncture. Immediately after blood collection, and while the animal remained under deep anesthesia, euthanasia was swiftly performed by cervical dislocation. This sequence ensured the acquisition of high-quality serum from a perfused circulation. Serum was separated by centrifugation at 3000× *g* for 10 min and stored at −80 °C until analysis. The organ index was calculated by quickly removing the liver, kidney, lung, thymus, and brain. In addition, liver, colon, and fecal tissues were collected, immediately flash-frozen in liquid nitrogen, and stored at −80 °C. This study was approved by the Scientific Ethics Committee of Lanzhou University (Ethics number: IRB24051601), and all procedures were performed in accordance with the Lanzhou University guidelines for the use of laboratory animals.

### 2.4. Biochemical Index Analysis

For the measurement of oxidative stress-related parameters in liver tissues, the liver samples were first weighed and homogenized in ice-cold normal saline (0.9% NaCl) at a weight-to-volume ratio of 1:9 (*w*/*v*) to prepare for 10% liver homogenate. The homogenate was centrifuged at 3000× *g* for 15 min at 4 °C, and the resulting supernatant was carefully collected for subsequent assays. The levels of interleukin-1β (IL-1β), interleukin-6 (IL-6), tumor necrosis factor-α (TNF-α), and interleukin-10 (IL-10) in the mice serum were detected using ELISA kits (Shanghai Kexing Trading Co., Ltd., Shanghai, China, catalog number: H002-1-2, H009-1-2, H052-1-2, H009-1-2). The activities of superoxide dismutase (SOD), glutathione (GSH), catalase (CAT), total antioxidant capacity (T-AOC), and malondialdehyde (MDA) in the liver tissues were measured using bio-chemical kits (Nanjing Jiancheng Biotechnology Institute, Nanjing, China, catalog number: A001-3-2, A005-1-2, A007-1-1, A015-2-1, A003-1-2).

### 2.5. Histopathological Analysis

Fresh mouse liver and colon tissues were first fixed in 4% paraformaldehyde (PFA) dissolved in phosphate-buffered saline (PBS, pH 7.4) for 24 h at 4 °C. Then the tissues were processed by gradient dehydration (70%, 80%, 90%, 95%, 100% ethanol), xylene clearing and paraffin embedding, and sectioned into 5 μm thick slices. These sections were stained with hematoxylin and eosin (H&E) staining kit (Nanjing Jiancheng Biotechnology Institute, Nanjing, China, catalog number: D006-1-1) following the manufacturer’s protocol. Stained sections were photographed and examined under a vertical microscope (Unioco Instrument Co., Ltd., Shanghai, China) for histopathological analysis.

### 2.6. Quantitative Real-Time Polymerase Chain Reaction (qPCR) Test

The qPCR test was conducted with modifications according to the procedure previously described by Liu et al. (2024) [16]. Briefly, total RNA was extracted from colon tissues stored at −80 °C using RNA Extraction Kit (Nanjing Jiancheng Biotechnology Institute, Nanjing, China, catalog number: N066). Tissue samples were ground in liquid nitrogen, and total RNA was isolated following the manufacturer’s protocol. The concentration and purity of RNA were verified using a NanoDrop 2000 spectrophotometer (Thermo Fisher Scientific, Waltham, MA, USA). cDNA synthesis was performed using the PrimeScript RT Reagent Kit (Takara Biomedical Technology Co., Ltd., Beijing, China, catalog number: RR037A) to eliminate genomic DNA contamination. Relative mRNA expression levels were quantified via qPCR on a LightCycler 96 instrument (Roche, Basel, Switzerland). Glyceraldehyde-3-phosphate dehydrogenase (GAPDH) was used as the house-keeping gene for normalization. The qPCR reaction was carried out with TB Green^®^ Premix Ex Taq™ II FAST qPCR (Takara, catalog number: RR830B) following standard cycling conditions (initial denaturation at 95 °C for 30 s, 40 cycles of 95 °C for 5 s and 60 °C for 30 s). Relative expression levels were calculated using the 2^−ΔΔCt^ method. The gene-specific primers used are shown in Table 1.

### 2.7. DNA Extraction and rRNA Gene Sequencing

The total genomic DNA of the fecal samples was extracted using TGuide S96 Magnetic Soil/Stool DNA Kit according to the instructions, and the extracted DNA was electrophoresed on 1.8% agarose gel, and the concentration and purity of DNA were determined by UV–visible spectrophotometer. The V3-V4 highly variable region of the bacterial 16S rRNA gene was amplified using primer pairs 338F: 5′-CTCCTACGGGGAGGCAGCA-3′ and 806R: 5′-GGACTACHVGGGGTWTCTAAT-3′. PCR amplification products were detected on agarose gels and purified by Omega DNA purification kits and quantified by Qsep-400. 400 for quantification. After purification and quantification, the amplicon libraries were subjected to paired-end sequencing on the Illumina novaseq6000 platform. Firstly, the quality of the data was assessed by filtering the raw data from using Trimmomatic v0.33, and then the primer sequences were identified and removed using cutadapt 1.9.1 software. After that, it was used for denoising, bipartite splicing and removal of chimeric sequences using the DADA2 method to obtain the final valid data. Alpha analysis was performed using QIIME2 software (version 2023.9) to determine the complexity of the species diversity of each sample. Beta Diversity calculations were performed using Non-Metric Multi-Dimensional Scaling (NMDS) to assess the species complexity of the samples. One-way ANOVA was used to compare bacterial abundance and diversity. Linear Discriminant Analysis (LDA) Effect Size (LEfSe) was used to assess the differential abundance taxa among groups. Bioinformatics analyses for this study were performed using the online platform BMKCloud (http://www.biocloud.net [accessed on 18 July 2025]).

### 2.8. Determination of Short-Chain Fatty Acids (SCFAs)

The method of Si et al. (2021) was adopted with slight modifications for the determination of SCFAs in mouse feces [17]. Briefly, frozen fecal samples were thawed at room temperature, and 50 mg of each sample was weighed into a centrifuge tube. After adding 400 μL of distilled water, the mixture was homogenized using a tissue grinder (SCIENTZ-48, Ningbo Xinzhi Co., Ltd., Ningbo, China) at 60 Hz for 1 min, then allowed to stand at 4 °C for 1 h, and centrifuged at 12,000× *g* for 15 min at 4 °C to collect the supernatant. A total of 0.02 mL of internal standard solution (2-ethylbutyric acid (2-EB) at a concentration of 0.2 g/L in 25% metaphosphoric acid deproteinization solution) was added to 0.2 mL of the collected supernatant, followed by thorough mixing, ice bath for 30 min, and centrifugation at 15,000× *g* for 15 min at 4 °C. The resulting supernatant was filtered through a 0.22 μm filter membrane before instrumental analysis. Samples were detected using a GC system (7890A, Agilent Technologies, Inc., Santa Clara, CA, USA) equipped with a 30 m × 0.32 mm × 0.35 μm HP-FFAP capillary column (8890-7000D, Agilent Technologies, Inc., Santa Clara, CA, USA). The GC operating conditions were as follows: injection volume of 0.6 μL; split ratio of 40:1; nitrogen as the carrier gas with a flow rate of 2.1 mL/min; injector temperature set at 250 °C; auxiliary box temperature at 250 °C; column oven temperature program: initial hold at 90 °C for 1 min, increased to 120 °C at a rate of 10 °C/min, then raised to 150 °C at 10 °C/min, and held for 3 min.

### 2.9. Statistical Analysis

The results were expressed as mean ± standard deviation (number of samples tested n ≥ 6, triplicate technical replications). Statistical analysis was performed using SPSS 25.0 software. A one-way ANOVA was used to determine significant differences among groups. Statistical significance was defined as *p* < 0.05. Correlations between gut microbiota and other variables were assessed using Spearman’s correlation test. The resulting *p*-values were subjected to stringent multiple testing correction via the Benjamini–Hochberg false discovery rate (FDR) procedure, and a corrected *p* < 0.05 was considered statistically significant.

## 3. Results

### 3.1. QL01 Inhibited Organ Atrophy in D-Galactose-Induced Ageing Mice

During ageing, various organs undergo atrophy as the animal’s weight increases, leading to a decrease in organ index. Therefore, changes in the organ index of senescent mice can be used as a valuable indicator when evaluating induced senescence models. As shown in Table 2, the MC group exhibited lower organ indices than the NC group, particularly in the thymus, liver, and kidney (*p* < 0.05). Importantly, the thymus, brain, and lung indices were significantly increased in the QL01 group (*p* < 0.05).

### 3.2. QL01 Regulated the Levels of Inflammatory Factors in D-Galactose-Induced Ageing Mice

**Figure 2:**
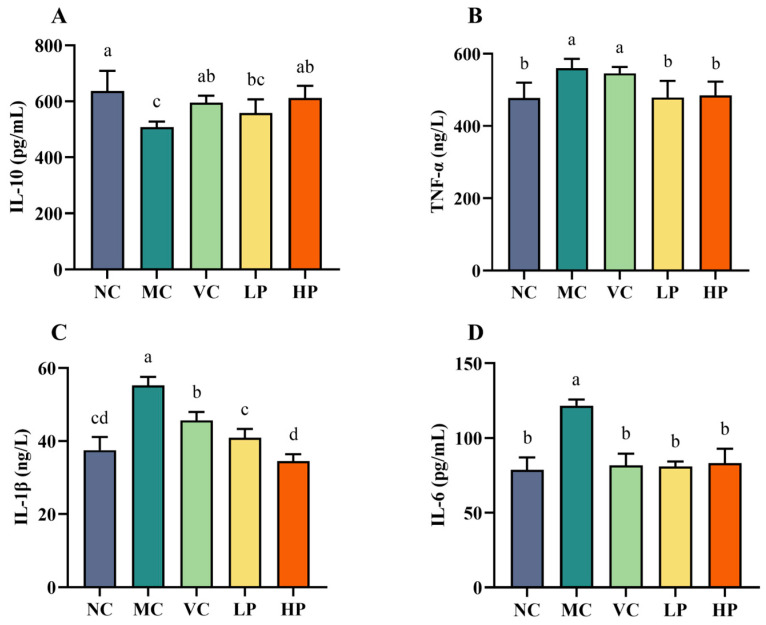
*L. plantarum* QL01 regulated inflammation responses induced by D-galactose-induced ageing mice. The concentration of IL-10 (**A**), TNF-α (**B**), IL-1β (**C**), and IL-6 (**D**) in the serum. Data are presented as mean ± SD (n = 6). (a–d) Mean values with different letters in the same bar graph are significantly different (*p* < 0.05).

Aging is associated with reduced immune function and increased inflammation. *Lactobacilli* are valued for their ability to modulate inflammatory markers. The present study therefore examined the serum levels of IL-6, IL-1β, TNF-α and IL-10 in each group of mice, to assess QL01’s ability to attenuate the inflammatory response in senescent mice. Serum levels of IL-6, IL-1β, TNF-α, and IL-10 in each group were measured to evaluate the potential of QL01 to attenuate the inflammatory response in ageing mice (Figure 2). The MC group exhibited significantly higher levels of TNF-α, IL-1β, and IL-6 after D-galactose administration. At the same time, IL-10 was markedly decreased (*p* < 0.05). This suggested that D-galactose induced an inflammatory response in the ageing mice. Compared to the MC group, both VC and QL01 treatments notably reversed these changes, with the high-dose of QL01 showing the most significant effect on IL-1β levels. These findings indicated that VC and QL01 could effectively inhibit D-galactose-induced expression of inflammatory factors in ageing mice.

### 3.3. QL01 Alleviated Hepatic Damage in D-Galactose-Induced Ageing Mice

**Figure 3:**
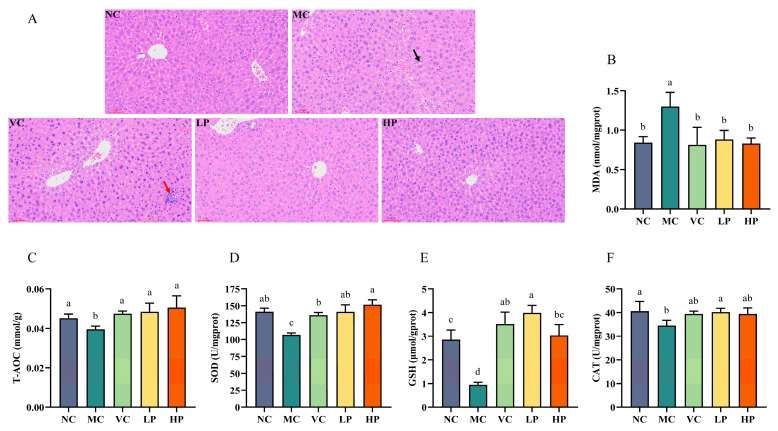
*L. plantarum* QL01 alleviated liver injury and oxidative damage in ageing mice. (**A**) Liver section analysis using hematoxylin-eosin staining. Scale bar: 60 μm. Magnification: ×200. Black arrow: fatty vacuole. Red arrow: inflammatory infiltration. (**B**) MDA levels in the liver. (**C**) T-AOC levels in the liver. (**D**) SOD levels in the liver. (**E**) GSH levels in the liver. (**F**) CAT levels in the liver. Data are presented as mean ± SD (n = 6). (a–d) Mean values with different letters in the same bar graph are significantly different (*p* < 0.05).

Excess D-galactose has a significant effect on hepatic antioxidant biomarkers because it is mainly metabolized in the liver. This is characterized by elevated levels of MDA in liver tissue, alongside reduced levels of antioxidant enzymes. Consequently, these biomarkers are frequently employed to evaluate the level of oxidative stress in the liver. Figure 3A illustrates the histopathological differences in the liver across the different groups. In the NC group, the hepatocytes appeared to have normal morphology, clear cell borders, prominent nucleoli, and regular arrangement. In contrast, hepatocytes from the MC group mice exhibited significant structural disorganization. For example, fatty vacuoles were present around blood vessels (black arrow), the boundaries between adjacent cells were blurred, and a few inflammatory cells were also observed. After VC and QL01 interventions, the fatty vacuolar injury in the mice disappeared. The VC group still showed signs of inflammatory infiltration (indicated by the red arrow). In contrast, the hepatocytes in the QL01 group were arranged in an orderly manner with clear borders and with reduced inflammatory infiltration. The results indicated that QL01 effectively improved D-galactose-induced hepatocyte damage.

### 3.4. QL01 Ameliorated Oxidative Damage in D-Galactose-Induced Ageing Mice

The biomarkers T-AOC, SOD, CAT, GSH, and MDA are commonly used to assess liver antioxidant status. As shown in Figure 3B, the liver tissues of mice in the MC group exhibited a significant rise in MDA content compared with the NC group (*p* < 0.05). The increasing MDA content suggested the presence of oxidative stress. In addition, D-galactose intervention led to a significant decrease in the concentrations of these four antioxidant enzymes (*p* < 0.05) (Figure 3C–F). This was successfully reversed by both the VC and QL01 interventions, indicating their protective effects. The results indicated that QL01 could protect the liver from D-galactose damage by activating the antioxidant enzyme system.

### 3.5. QL01 Restored Intestinal Homeostasis in D-Galactose-Induced Ageing Mice

**Figure 4:**
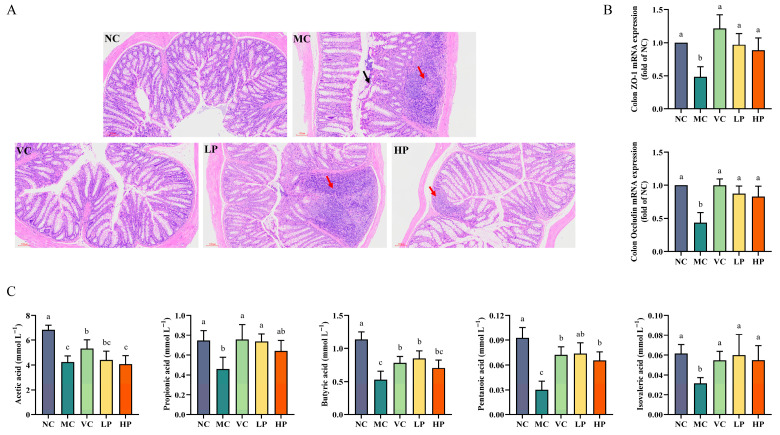
*L. plantarum* QL01 restored intestinal homeostasis in D-galactose-induced ageing mice. (**A**) The colon sections were stained with hematoxylin-eosin. Scale bar: 100 μm. Magnification: ×40. Black arrow: the goblet cells were severely damaged. Red arrow: inflammatory cells. (**B**) The mRNA levels of ZO-1 and Occludin in the colon tissue of mice. (**C**) The levels of SCFAs in the feces for each group. Data are presented as mean ± SD (n = 6). (a–c) Mean values with different letters in the same bar graph are significantly different (*p* < 0.05).

Histological analyses were performed using H&E staining to assess the impact on the colon. The observations showed that the integrity of the intestinal barrier was compromised in the MC group (red arrow) in comparison with the NC group. A significant number of inflammatory cells were observed in the basal layer, and intestinal mucosal epithelial cells were shed, leaving portions of the intestinal mucosa absent. Additionally, the goblet cell sites are severely damaged (black arrow). The treatment of high-dose QL01 was effective compared to the LP group, with a significant reduction in inflammatory cells, normalization of the intestinal mucosa, and preservation of the basal layer structure (Figure 4A). Furthermore, it was found that ZO-1 and Occludin expression levels decreased significantly in ageing mice by analyzing tight junction proteins mRNA levels. However, the QL01 treatment caused them to become significantly upregulated (*p* < 0.05) (Figure 4B). In addition, the SCFAs levels were significantly lower in the MC group. In contrast, QL01 resulted in a significant increase in SCFAs levels (*p* < 0.05) (Figure 4C). These results indicated that QL01 restored intestinal homeostasis in ageing mice.

### 3.6. QL01 Modulated the Composition of Gut Microbiota

**Figure 5:**
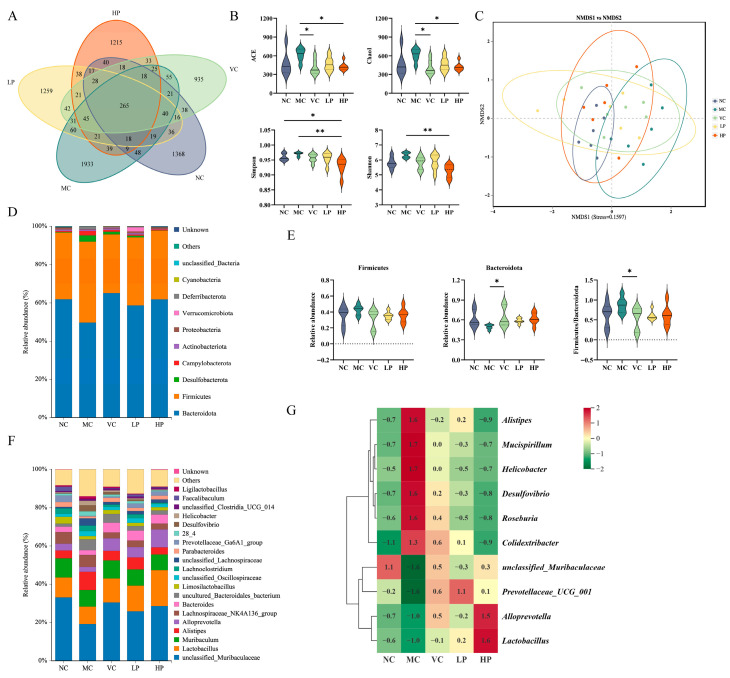
The change of gut microbiota in D-galactose-induced ageing mice with the presence of *L. plantarum* QL01. (**A**) Venn diagram for each test group. (**B**) Violin plot of alpha diversity illustrates the diversity of each group. (**C**) Beta diversity analysis of intestinal microbiota using the NMDS method. (**D**) The difference in the microbial community at the phylum level. (**E**) The relative abundances of Firmicutes and Bacteroidota in different groups. (**F**) The difference in the microbial community at the genus level. (**G**) Heatmap of the relative abundance of significantly different bacteria in the intestinal flora at the genus level in each group. All data are indicated as mean ± SD of 6 mice per group. * represents *p* < 0.05; ** represents *p* < 0.01.

To better investigate the regulatory effects of QL01 on the intestinal flora of senescent mice, the diversity, abundance, composition and community structure of mouse intestinal flora were analyzed by 16S rRNA gene sequencing. Visualize the similarities and differences among all the samples using Venn diagrams. A total of 265 operational taxonomic units (OTUs) were found across the five groups. The MC group exhibited the highest number of unique taxa with 1933 OTUs, followed by the LP group (1259 OTUs), the HP group (1215 OTUs), the NC group (1368 OTUs), and the VC group (935 OTUs) (Figure 5A). Figure 5B showed alpha diversity in five groups, including ACE, Chao 1, Simpson, and Shannon indices. These indices offered distinct insights into the gut microbiota’s diversity and richness. Although the differences were not statistically significant, the MC group showed a slight increase in all four indices compared with the NC group (*p* > 0.05). The HP group significantly reduced the diversity index compared with the MC group (*p* < 0.05). This suggested that QL01 could significantly decrease gut microbiota diversity and richness, highlighting its potential to alter the gut microbiota in mice.

Beta diversity analysis, aided by non-metric multidimensional scaling (NMDS), provided valuable insights into comparison of gut microbiota composition across groups. As shown in Figure 5C, the sample points of the normal control (NC) and model control (MC) groups exhibited limited overlap, suggesting that D-galactose may alter the composition of the gut microbiota. Among the intervention groups, the HP group showed greater overlap with the MC group compared to the VC and LP groups, indicating that supplementation with QL01 may help shift the gut microbiota structure of aging mice toward a normal state. These results imply that QL01 could have a beneficial modulatory effect on the gut microbiota composition in aging mice.

We compared the distinct community profiles of each group at different taxonomic levels to further explore the compositional diversity of the gut microbiota. As depicted in Figure 5D, the most prevalent microbial phyla among the five groups of mice were Bacteroidota and Firmicutes. Compared with the NC group, the abundance of Bacteroidetes in D-galactose-induced mice decreased from 61.88% to 49.61%. This shift was accompanied by an increase in the Firmicutes percentage from 34.88% to 42.36% and an increase in the F/B ratio. However, the intervention of Vit C and QL01 mitigated these changes, and the improvement of VC was significant (*p* < 0.05) (Figure 5E).

At the genus level, the five groups showed a high relative abundance of *unclassified_Muribaculaceae*, *Lactobacillus*, *Muribaculum*, *Alistipes*, and *Alloprevotella* (Figure 5F). A heat map displaying the relative abundance of bacteria with significant differences in the gut microbiota of each group of mice was provided in Figure 5G. D-galactose treatment caused a significant decrease in the relative abundance of unclassified_*Muribaculaceae*, *Prevotellaceae_UCG_001*, *Lactobacillus*, and *Alloprevotella* compared with the NC group. However, other gut microbiota genera, including *Mucispirillum*, *Helicobacter*, *Alistipes, Desulfovibrio*, *Roseburia*, and *Colidextribacter*, showed the opposite trend, with significant increases in abundance in the MC group (*p* < 0.05). Their levels could be restored by supplementation with Vit C and QL01, particularly at high doses of QL01. These findings suggested that gavage with QL01 restored the D-galactose-induced imbalance in the gut microbiota.

### 3.7. Correlation Analysis of Gut Microbiota with Biochemical Parameters and SCFAs

**Figure 6:**
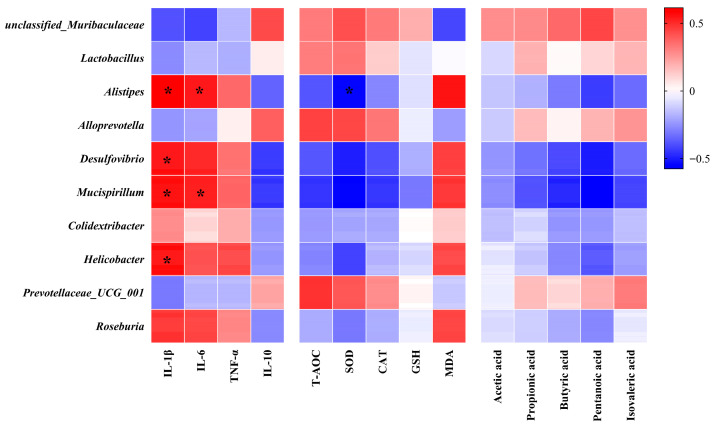
Spearman’s correlation analysis of gut microbiota with inflammatory factors, antioxidant enzyme levels, and SCFAs (n = 6). The color intensity shows the strength of the correlation, * indicates *p*-value (FDR-adjusted *p*-value) < 0.05. The red color represents a positive correlation, whereas the blue color represents a negative correlation.

We performed Pearson correlation analyses to examine associations among bacterial taxa, inflammatory factors, biomarkers of oxidative stress, and SCFAs. *Alistipes*, *Desulfovibrio*, *Mucispirillum*, *Helicobacter*, and *Roseburia* were significantly positively correlated with pro-inflammatory factors, but inversely correlated with antioxidant enzymes and SCFAs, as shown in Figure 6. However, unclassified_*Muribaculacea*, *Prevotellaceae_UCG_001*, and *Alloprevotella* were positively correlated with anti-inflammatory factors and antioxidant enzymes.

## 4. Discussion

Functional foods with therapeutic benefits are increasingly sought by contemporary consumers, with one of the main goals being the protection of the body from the effects of ROS [18]. To achieve this, it is necessary to explore natural antioxidants that promote health. Probiotics have been recognized as a promising source of antioxidants, particularly amid the growing emphasis on microecological dietary supplements [19]. Continuous injection of D-galactose is known to increase ROS and decrease antioxidant enzyme activity in mice, ultimately leading to heightened oxidative stress and accelerated ageing [20]. Therefore, in this study, the in vivo antioxidant effects of *L. plantarum* QL01 were investigated using the D-galactose-induced ageing model.

During ageing, various organs undergo shrinkage, leading to a decrease in organ index. Therefore, changes in the organ index of ageing mice can serve as valuable indicators for evaluating the induction of ageing models [21]. Organ indices were also used to express changes in the ageing degree in the studies by Li et al. (2020) and Zhou et al. (2021) [22,23]. The current study confirmed that D-galactose induced organ atrophy in mice. QL01 effectively restored this alteration, resulting in organ indices remaining like those of normal mice. These findings indicated that QL01 promoted organ health in ageing mice, providing a protective effect. Furthermore, ageing is associated with reduced immune function and increased inflammation, and probiotics are emphasized for their regulatory effects on inflammatory markers [24]. Our results showed that D-galactose stimulated the production of inflammatory factors, promoting inflammatory responses. In contrast, serum anti-inflammatory cytokine levels increased, and pro-inflammatory cytokine levels decreased after the QL01 intervention. These findings were like those observed with pretreatment using *Bacillus coagulans* JA845 [25]. These results confirmed that the QL01 intervention not only has the potential to protect organs but also helps reduce neuroinflammation.

As D-galactose is primarily metabolized in the liver, excessive D-galactose significantly affects hepatic antioxidant biomarkers. This effect was characterized by increased levels of MDA and decreased levels of antioxidant enzymes (including T-AOC, CAT, SOD, GSH, and GSH-Px) in liver tissue. These changes induce oxidative stress in the liver [26]. However, in this work, it was observed that QL01 treatment significantly reduced MDA levels in the liver tissue. Increased activities of T-AOC, CAT, SOD, and GSH accompanied this. Furthermore, liver histopathology provided insights into the extent of oxidative damage induced by oxidative stress. Therefore, the results suggested that QL01 may alleviate D-galactose-induced oxidative damage in the liver by enhancing antioxidant defense mechanisms. The results of Wang et al. (2023) supported our study, suggesting that probiotic intervention could enhance the liver’s antioxidant capacity to combat oxidative stress [12].

The integrity and function of the intestinal barrier are compromised, characterized by increased permeability of epithelial tight junctions, damage to goblet cells, and the onset of inflammation during the ageing process [27]. Consistently, these impairments of the gut barrier were also observed in our study. Probiotics may increase TJP production, potentially aiding in restoring the gut barrier. For example, *Lactobacillus plantarum* NK3 has been demonstrated to reduce permeability and enhance barrier function in animal models [28]. In line with these results, our study showed that QL01 increased ZO-1 and Claudin-1 gene expression, increased goblet cell numbers, and reduced inflammation in intestinal sections. These findings indicated that QL01 enhanced barrier function.

It is well known that ageing is associated with changes in the composition and function of the gut microbiota, commonly referred to as dysbiosis. Probiotic regulation with *Lactobacillus* presents a promising therapeutic approach [29]. Our analysis of the gut microbiota revealed increased alpha diversity in the MC group. While some studies aligned with our findings, others suggested that diversity decreases in ageing models [1]. This highlighted that the gut microbiota was a complex and extensive system [30]. The intervention with QL01 did not significantly change the structure and composition of the gut microbiota. However, there were significant differences in levels of specific gut microbes across groups. These specific shifts in microbes may help counter oxidative stress and inflammatory responses associated with ageing [31].

In the mouse gut microbiota, the two most dominant phyla are Bacteroidota and Firmicutes. The MC group showed a reduction in Bacteroidota abundance and an increase in Firmicutes abundance, resulting in a higher F/B ratio. As in previous studies, ageing was associated with an enrichment of Firmicutes [32]. However, some studies reported the opposite findings, and these discrepancies may be due to variations in specific Firmicutes members [33,34]. Interestingly, *L. plantarum* belongs to the Firmicutes; intervention with QL01 reversed changes in the F/B ratio, suggesting it may have a positive influence on ageing [35]. A more detailed analysis revealed that the abundance of beneficial bacteria, including *Lactobacillus* and *Alloprevotella*, increased in the probiotic group, particularly in the HP group, where *Lactobacillus* was significantly enriched. This indicated that QL01 successfully colonized the mouse gut and expanded the resident microbiota [36]. Studies have reported that *Lactobacillus* plays a role in regulating the body’s stress levels and in alleviating the effects of ageing [37]. Thus, the ameliorative effect of QL01 on oxidative stress in this study might significantly contribute to the enrichment of *Lactobacillus* [38]. Additionally, the increase in *Alloprevotella* aligned with previously observed results [16]. *Alloprevotella* is linked to improvements in human mood and has been found to increase mucin synthesis while reducing intestinal permeability [39]. In addition, *Lactobacillus plantarum* H6 effectively enhanced the abundance of unclassified Muribaculaceae, consistent with our results. Notably, a higher proportion of potential pathogens, including *Helicobacter*, *Colidextribacter*, and *Desulfovibrio*, were observed in ageing model mice. *Helicobacter* are inflammation-related bacteria. Reports indicate that Helicobacter negatively affects cognitive function in mice with Alzheimer’s disease [40]. The increased abundance of *Colidextribacter* is linked to impaired intestinal barrier function. Likewise, *Desulfovibrio* is closely associated with increased intestinal permeability and host inflammatory responses [41]. Further correlation analysis showed that the abundance of pathogenic bacteria was significantly positively correlated with pro-inflammatory factors, suggesting that QL01 may prevent D-galactose-induced immune disorders by inhibiting intestinal pathogens such as *Desulfovibrio*. In short, QL01 is an effective probiotic to restore the intestinal microbial community.

Probiotics’ regulation of intestinal microbial metabolites is considered beneficial. SCFAs, primarily acetate, propionate, and butyrate, play important roles in host energy metabolism, regulation of inflammation, and antioxidant activity [42]. The number of bacteria that produce short-chain fatty acids in the gut decreases with age, leading to lower levels of these fatty acids [43]. Our findings support this idea. The increased SCFA concentration in the QL01 group may be due to the accumulation of strain in the gut. The strain’s strong survival and colonization abilities in the gastrointestinal tract likely help regulate the gut microbiota by stabilizing SCFAs-producing bacterial populations and enhancing SCFA production (Yin et al., 2024) [43]. Thus, the regulation of SCFA production represented a potential benefit of supplementing with QL01. In brief, we proved the in vivo antioxidant effects of *L. plantarum* QL01. The results showed that QL01 could boost the host’s antioxidant capacity and help to maintain intestinal flora homeostasis and metabolism.

## 5. Conclusions

In conclusion, our research provides strong evidence for the beneficial effects of *Lactobacillus plantarum* QL01 in alleviating oxidative stress and delaying ageing. The beneficial effects of QL01 supplementation encompassed multiple aspects: the inhibition of organ atrophy, modulation of serum inflammatory factor levels, enhancement of hepatic antioxidant enzyme activity, and increased colon levels of Claudin-1 and ZO-1. In addition, modulation of the gut microbiota, specifically elevated abundance of beneficial bacteria, reduced abundance of pathogenic bacteria, and increased levels of SCFAs, were demonstrated to contribute to these favorable outcomes. Thus, *Lactobacillus plantarum* QL01 exhibits considerable potential as a safe, eco-friendly, and high-quality probiotic strain with antioxidant capacities.

## Figures and Tables

**Table 1:** Genes and Primers selected for qPCR.

Gene	Primer	Sequence (5′ to 3′)
ZO-1	forward	AAGCAGTGGAAGAAGTTACAGTTGAG
	reverse	TTGAGCATACACAGGTTTCGGTTC
Occludin	forward	TTGAAAGTCCACCTCCTTACAGA
	reverse	CCGGATAAAAAGAGTACGCTGG
GAPDH	forward	AGGTCGGTGTGAACGGATTTG
	reverse	GGGGTCGTTGATGGCAACA

**Table 2:** Effects of *L. plantarum* QL01 on organ indices of ageing mice induced by D-galactose.

Groups	Organ Index (g/g) %				
	Thymus	Brain	Liver	Kidney	Lung
NC	0.195 ± 0.034 *	1.088 ± 0.074	3.585 ± 0.135 *	1.327 ± 0.077 *	0.575 ± 0.062
MC	0.153 ± 0.013	0.986 ± 0.070	3.339 ± 0.288	1.139 ± 0.088	0.530 ± 0.022
VC	0.188 ± 0.032	1.084 ± 0.056	3.495 ± 0.200	1.334 ± 0.197 **	0.575 ± 0.061
LP	0.194 ± 0.021 *	1.111 ± 0.094 *	3.373 ± 0.104	1.225 ± 0.095	0.576 ± 0.030 *
HP	0.205 ± 0.038 **	1.100 ± 0.098 *	3.516 ± 0.151	1.235 ± 0.088	0.620 ± 0.069 *

NC: normal control group, MC: model control group, VC: positive control group, LP: low-dose QL01 group, and HP: high-dose QL01 group. Compared with MC, * represents *p* < 0.05; ** represents *p* < 0.01.

## Data Availability

Data will be made available on request.
